# Thermodynamic and Structural Insights into Endocrine Disruptor Adsorption

**DOI:** 10.1021/acs.jpcc.6c02106

**Published:** 2026-07-06

**Authors:** Sam Shepherd, Laura McWilliams, Oliver S. Cunningham, Helen Lubarsky, Gareth A. Tribello, Debra H. Phillips, David M. Wilkins

**Affiliations:** † Centre for Quantum Materials and Technologies, School of Mathematics and Physics, 1596 Queen’s University Belfast, Belfast, Northern Ireland BT7 1NN, United Kingdom; ‡ School of Natural and Built Environment, Queen&apos;s University Belfast, Belfast, Northern Ireland BT7 1NN, United Kingdom

## Abstract

Endocrine disrupting chemicals (EDCs) pose a significant threat to human health and to the environmentat very low concentrations due to their ability to mimic natural hormones. Adsorption-based removal of EDCs from water is an increasingly attractive decontamination strategy, yet the structural and chemical factors governing the binding of these molecules to common adsorbents remain poorly understood. In this work, we employ well-tempered metadynamics simulations to compute the free energies of adsorption for 12 EDCs on the surface of a single-walled carbon nanotube (SW-CNT). We find that binding free energies correlate broadly with the number of electrons, reflecting the importance of dispersion interactions, but that this relationship has several notable exceptions. Conformational flexibility is identified as an additional critical factor: molecules bearing two aromatic groups connected via a heteroatom (oxygen or nitrogen) can adopt coplanar configurations that maximize π-stacking interactions with the CNT surface, leading to substantially stronger binding. Conversely, sterically rigid molecules, regardless of the presence of aromatic groups or increased number of electrons, bind weakly to the CNT surface. These findings demonstrate that both the chemical composition and conformational flexibility of an EDC must be considered when evaluating the suitability of CNT-based adsorbents for water decontamination applications, as well as showing the importance of extensive computational studies as complementary to experiment.

## Graphical abstract

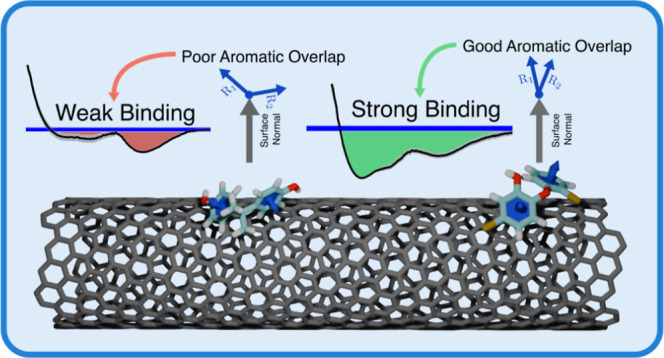

## Introduction

Endocrine disrupting chemicals (EDCs) are compounds that mimic naturally occurring hormones by binding with hormone receptors in the human body. Removing these compounds from the environment is one of the key challenges faced by researchers in the environmental and physical sciences.^1–6^ EDCs are commonly byproducts or metabolites from the degradation of other chemicals used in common products like plastics^7,8^ or personal care products.^9–13^ In other cases, chemicals which were developed without a full appreciation of their postapplication life cycle have been identified as possessing endocrine disrupting qualities. A key example of this can be found in agriculture. One of the first documented EDCs was the insecticide dichlorodiphenyltrichloroethane (DDT), which was found to interfere with the endocrine system of birds,^14–19^ mammalian wildlife,^16,20^ and humans^21–23^ from as early as 1947. Due to their ability to mimic hormones, EDCs pose a serious issue to human health even in small concentrations. They cause a number of severe developmental and reproductive health issues including reproductive defects,^24–26^ cancer,^27,28^ and developmental disorders.^4,29–31^ These issues are further exacerbated by the ability of EDCs to bioaccumulate in the body.^32–34^ Research on developing viable alternatives to chemicals which can metabolize into EDCs^35–37^ and efforts to reduce the level of EDCs in the environment are thus essential if we are to limit or halt the bioaccumulation process.^38–41^

Much of the research over the last 35 years has focused on removing endocrine disruptors from water^42–47^ in the hope that doing so will prevent them from bioaccumulating in plants and animals.^48–51^ There are multiple methods for removing contaminants from water. When deciding which method to use, one must consider the efficiency of the removal process, the rate at which it is removed, the production of toxic byproducts, the regeneration of any removing agent, and the cost.^52–54^ In addition to the wide range of possible methods available to remove these contaminants, there is also no guarantee that a method that works well for one contaminant transfers to another,^55,56^ particularly given that small changes in the structure of a contaminant can lead to large changes in its chemical and physical properties.^57–62^

A common method for removing contaminants from drinking water is to transform them into a less harmful form via processes such as oxidation,^63–66^ reduction,^67,68^ and photocatalysis.^54,56,69^ However, a large number of EDCs have low chemical reactivity under ambient conditions,^70–73^ and others have the potential to form toxic byproducts upon reaction.^70^ Because of these concerns, decontamination methods that are based on physically adsorbing the contaminant molecule to a substrate are becoming more popular.^39,41,54,55,74–79^ Materials such as graphene^80–82^ and other naturally sourced forms of carbon,^83–87^ zerovalent iron,^88–92^ and silica resin beads^93–96^ can all be used to remove particular contaminants from water. Carbon nanotubes are a particularly promising variety of carbon-based materials owing to their high surface area and tunable surface chemistry.^97–99^ Surface alterations, including hydroxylation of the surface,^100^ can also help in the targeting of particular organic contaminants. Ultimately, however, a single substrate that binds all the various EDCs has not been identified. In fact, identifying a suitable method for removing a particular EDC is challenging and requires a considerable outlay of human time.

Atomistic simulation is routinely used to explore the adsorption of organics on surfaces, including iron oxides,^101,102^ graphene and idealized carbon nanotubes,^103^ as well as mineral surfaces like kaolinite or zeolites.^104,105^ However, many of these studies have focused on studying the adsorption of one particular molecule on one particular substrate. There have been few systematic studies that compare how different molecules adsorb on the same substrate.^103^ The hope in performing such studies is that one can discover principles that can then be used to design decontamination strategies.

In this paper, we use molecular dynamics simulation to determine the free energies of adsorption for 12 EDCs on the surface of a single-walled carbon nanotube (SW-CNT), a substrate commonly used as an adsorbent in the water treatment industry.^97,106,107^ We show that the number of electrons in each molecule is a reasonably good predictor of the binding free energy. There are, however, some cases where molecules with similar sizes but different functional groups have very different binding free energies. Molecules that contain aromatic groups bind strongly when the aromatic groups can lie flat on the CNT surface and maximize π-stacking interactions. Conformational flexibility is thus critical for strong binding as flexibility allows molecules to adopt favorable binding configurations.

## Methods

The molecules that we study encompass a wide variety of chemical interactions, functional groups, and molecular structures. In total, we studied the adsorption of 12 organic molecules. All of these chemicals are listed as having some capacity to act as an endocrine disrupting chemical or are under evaluation.^108^ Table 1 lists each of the EDCs studied in this work, along with their full IUPAC names and molecular formulas. We note that we use the common names in place of the full IUPAC names for both ALM and PCZ.

**Table 1:** Acronyms, IUPAC Names, and Molecular Formulae of the 12 EDCs Studied in This Work^a^

acronym	name^a^	molecular formula
TCS	2,4,4′-Trichloro-2′-hydroxydiphenyl ether	C_12_H_7_Cl_3_O_2_
BPA	2,2-Bis(4-hydroxyphenyl)propane	C_15_H_16_O_2_
ALM	Asulam*	C_8_H_10_N_2_O_4_S
MBC	3-(4-Methylbenzylidene)camphor	C_18_H_22_O
PCZ	Propiconazole*	C_15_H_17_Cl_2_N_3_O_2_
MDP	4-(10-Methylundecyl)phenol	C_18_H_30_O
MDM	2-Pyrimidinamine, 4-methyl-*N*-phenyl-6-(1-propyn-1-yl)	C_14_H_13_N_3_
BOP	Butyl 4-hydroxybenzoate	C_11_H_14_O_3_
BPB	2,2-Bis(4-hydroxyphenyl)butane	C_16_H_18_O_2_
DBnPA	2,2-dibromo-3-nitrilopropionamide	C_3_H_2_Br_2_N_2_O
DPP	4-(2,3-Dimethyl-3-pentanyl)phenol	C_13_H_20_O
BBP	Benzyl butyl phthalate	C_19_H_20_O_4_

a Asterisks denote molecules where the common names have been used in place of their IUPAC names.

Figure 1(i) shows a schematic of a subset of these molecules around a CNT. The selected subset of molecules represents a suitably diverse yet concise range of chemical structures and functional groups to represent the broader family of molecules that possess endocrine disrupting capabilities.

**Figure 1:**
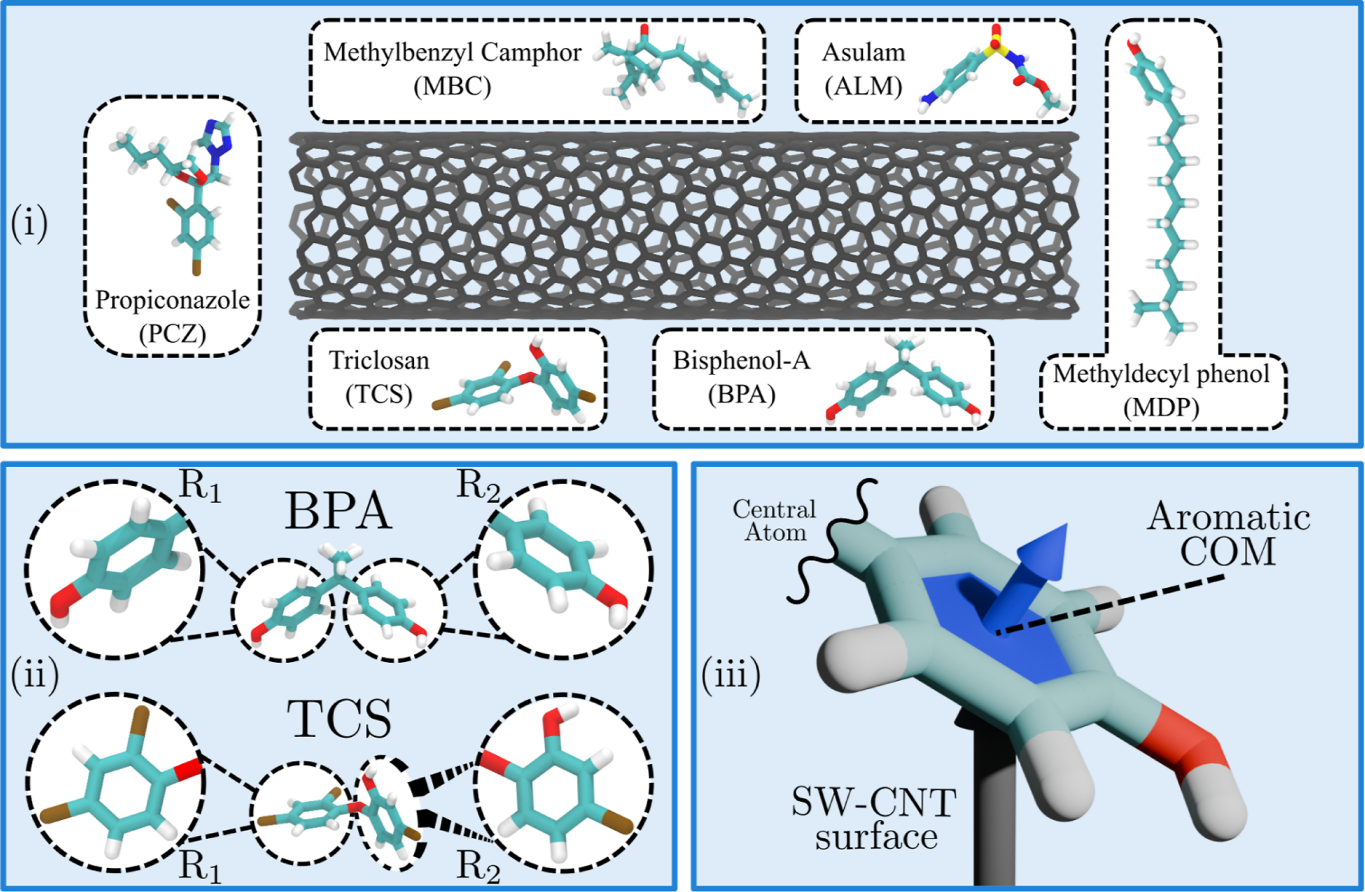
(i) Schematic figure of six EDCs discussed in this manuscript. An image of the molecules, as well as their common names and acronyms used in this work, are provided in the white boxes and the SW-CNT pictured in the center of the panel. Elements are colored as follows: white atoms are hydrogen, cyan atoms are carbon, red atoms are oxygen, yellow atoms are sulfur, dark blue atoms are nitrogen, and brown atoms are chlorine. (ii) Breakdown of the individual EDC molecules using the structural generalizations of this work. We use both BPA and TCS as examples to illustrate this breakdown into constituent R groups. (iii) Illustration of the vectors used to compute the orientation of the R groups on the SW-CNT surface.

### Molecular Dynamics Simulations

Each of the EDCs was studied using classical molecular dynamics (MD) simulations. All simulations were performed using LAMMPS (v29Aug2024).^109^ In these simulations, a CNT with chiral indices (15,5) and radius of 6.67 Å was placed in the center of a 50 × 50 × 62 Å^3^ box with the axis of the tube orientated parallel to the *z* axis. A CNT was selected because it is a commonly employed adsorbate in experimental studies regarding the removal of organic contaminants.^97,107,110^ In addition, the exterior surface of a CNT presents a chemically uniform environment which greatly simplifies a study of adsorption across a range of different organic molecules. A single EDC molecule was randomly placed inside the cell and outside of the CNT, and the remaining exterior space was filled with water to a standard density of ∼1 g/cm^3^. The interior of the nanotube was kept vacant. A schematic image of these systems is included in Figure 2 (panel (i)) with the water excluded for clarity.

**Figure 2:**
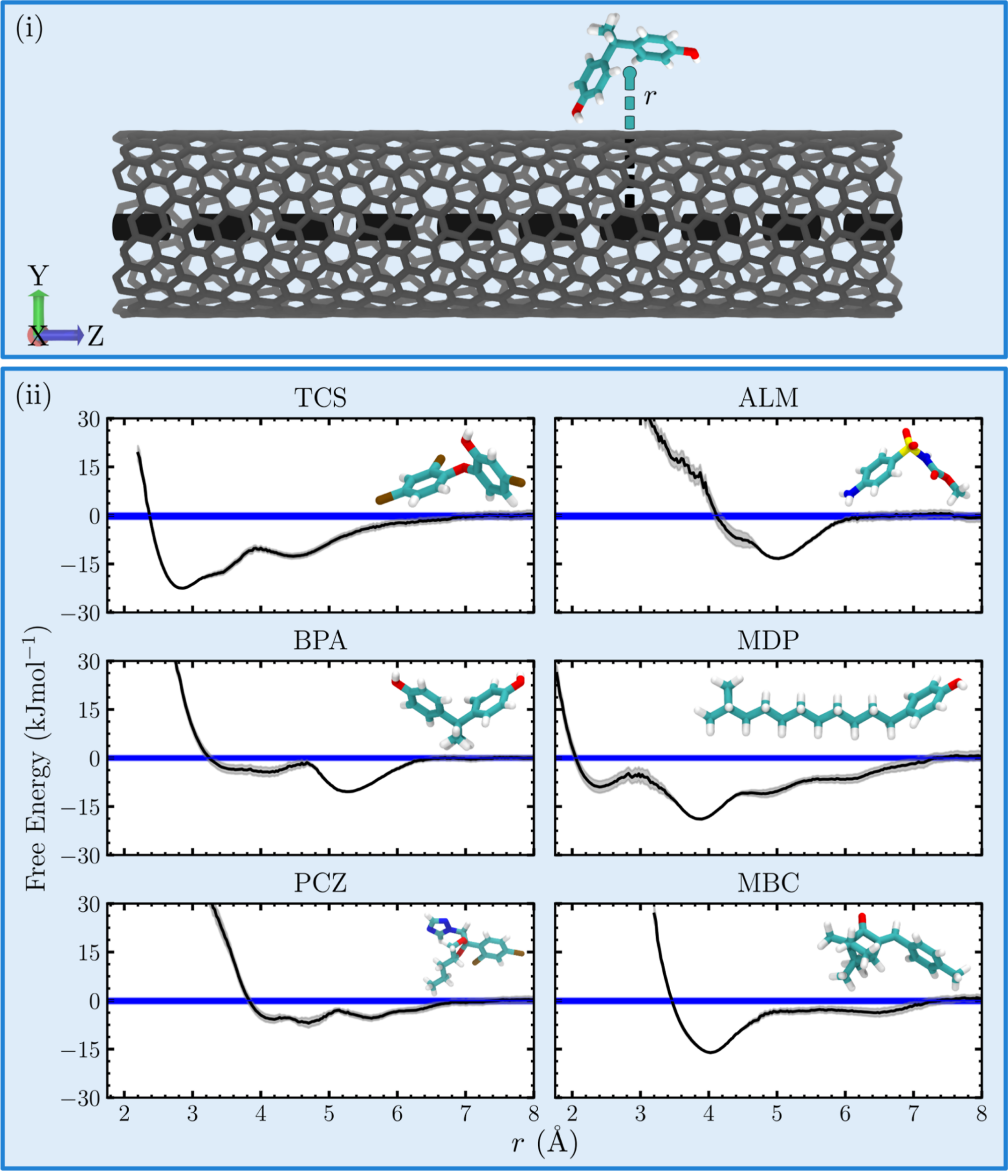
(i) Schematic image showing the simulation setup with BPA on the surface of the CNT. The large dashed black line in the CNT represents the COM projected along the length of the CNT. The small dashed black line and the dashed cyan line represented the radius of the CNT and the distance from the molecular COM to the surface of the CNT, denoted by *r*. (ii) Free energy as a function of distance from the surface of the CNT for a subset of six EDCs studied in this work. The error in the free energy profile is the gray area around the black line, while the error in our baseline is the lighter blue area around the solid blue line.

Input files for our simulations can be found at https://github.com/sshepherd637/EDCsOnStuff.

After setting up the systems, an energy minimization was performed with an energy convergence criteria of 1 × 10^–6^ kcal mol^–1^ and a force convergence criteria of 1 × 10^–8^ kcal mol^–1^ Å^–1^ per atom. Following the completion of this optimization procedure, a 5 ns NVT equilibration was performed, followed by 5 ns of NPT simulation. A Nose–Hoover thermostat was applied to the simulations and set to a target temperature of 298.15 K with a relaxation time of 500 fs. Similarly, an isotropic Nose–Hoover barostat was applied to the NPT simulations with a target pressure of 1 atm and a relaxation time of 1 ps. We note that an isotropic barostat was used in place of an anisotropic barostat (the recommended barostat for this system) to aid in the construction and analysis of the metadynamics simulations. All of the interactions between atoms were described by the OPLS-AA force field.^111–115^ We employed the SPC/E model for water,^116^ which was kept rigid using the SHAKE algorithm^117,118^ as implemented in LAMMPS. More computationally demanding methods, such as electronic structure theory, have been used to study the adsorption of organic molecules.^80^ For example, Fischer employed molecular dynamics (MD), density functional theory (DFT), and *ab initio* molecular dynamics (AIMD)^119,120^ to study the absorption mechanism of the endocrine disruptor triclosan on zeolites at the quantum-mechanical level. However, we want to perform large numbers of simulations with large numbers of atoms over long time scales. To do this with DFT would be too computationally expensive.

### Metadynamics

To find the free energy of adsorption of an EDC molecule onto the surface of the CNT, we used well-tempered metadynamics^121–125^ in which the distance *r* between the center of mass of the EDC and the surface of the CNT served as the collective variable (CV). This CV is illustrated in Figure 2, panel (i). To evaluate it, we calculate the distance between the center of mass of the molecule and the center of mass of the nanotube. We then subtract the average radius of the nanotube, which is evaluated as the average distance between the nanotube’s center of mass and the carbon atoms.

All metadynamics calculations were carried out using PLUMED (v2.6.6) and LAMMPS. These simulations were carried out in the *NPT* ensemble using the settings previously outlined. A harmonic restraint, *V*_wall_, with the following form

$$V_{wall}\left(r\right)=\{\begin{array}{ll} k|r-10.0{|}^{2} & r\geq 10Å,\\ k|r-0.05{|}^{2} & r\leq 0.05Å, \end{array}$$

and spring constant *k* = 4000 kJ/Å^2^ was also applied on the CV. A bias factor of 7 was used in the metadynamics calculations and Gaussians with a width of 1.0 Å and a height of 1.2 kJ mol^–1^ every 100 time steps. All simulations were run for a minimum of 10 ns.

We also provide input files for an example metadynamics calculation in our GitHub repository.

## Results and Discussion

### Free Energy Calculations

The free energy as a function of the distance between the center of mass of the molecule and the CNT was extracted from our metadynamics simulations by reweighting. Errors on the estimates of these quantities were obtained by constructing 6 separate estimates of the free energy from each 1.5 ns segment of the trajectory and computing the standard deviation between these 6 estimates.

If there is no interaction between the molecule and the CNT, the free energy as a function of the distance between the center of mass of the CNT and the molecule is given by

1 $$f\left(r\right)=-k_{B}T\;\ln\left(r\right)+c$$

where *k*_B_ is Boltzmann’s constant, *T* is the temperature, and *c* is a fitting parameter.

Consequently, some further analysis of the free energy surfaces that emerge from the metadynamics simulation is performed before they are reported in Figure 2. We assume that there is no interaction between the CNT and the molecule once they are more than 7 Å apart and thus find *c* in the expression as the value which minimizes the root-mean-square error

$$RMSE=\sqrt{\frac{\sum_{i=1}^{N}|g_{i}+k_{B}T\;\ln\left(r_{i}\right)-c{|}^{2}}{N}}$$

where *g*_*i*_ is the block-averaged value of the free energy from our metadynamics simulation for the bin centered on *r*_*i*_ and where *N* encompasses all the points on the grid between 7 and 8 Å. The bin width was set to 0.025 Å, and hence, *N* has a value of 40. This range starts at a point where the interaction between the CNT and the molecule would be expected to be minimal and ends before the additional wall potential takes effect. The free energy surfaces in Figure 2 show the difference between the free energies that are obtained from metadynamics and eq 1 for six of the studied EDCs. The remaining six free energy profiles are presented as Figure S2 in the Supporting Information. The baselining procedure incurs an additional source of error due to the standard deviation of the free energy profile between 7 and 8 Å. This error is illustrated by the blue shaded area within the free energy surfaces of Figures 2 and S2 and represents the maximum standard deviation between the 6 blocks in the range of 7–8 Å. The value of this curve at the point *r* gives the difference in free energy between a system where the molecule and CNT are *r* apart and a reference state in which noninteracting versions of the CNT and molecule are separated by the same distance. We thus use the following integral:

$$I=-k_{B}T\;\ln\left[\int_{r_{finite}}^{r_{\max}}e^{-\left(g\left(r\right)-k_{B}T\;\ln\left(r\right)+c\right)/k_{B}T}dr\right]$$

as a measure of the strength of the interaction between the molecules and the CNT surface. Defining this common reference state for all the molecules allows us to compare the binding free energies for the various molecules like that shown in Figure 2. This integral was computed numerically using the trapezoid rule with a bin width of 0.025⁡Å . The bounds of the integration were located at the point *r* in which *g*(*r*) became finite (lower bound) and r=8⁡Å .

Figure 3 shows the binding free energy for each of the molecules as a function of the number of electrons in the molecule (panel (i)) and the distance from the molecular center of mass to the CNT surface (panel (ii)). Panel (i) shows that the strength of the dispersion interactions is proportional to the number of electrons. This means that molecules with higher electron counts would be expected to bind more tightly to the CNT surface. The general trend in Figure 3i follows this pattern, but with some notable exceptions. The most significant of these exceptions is PCZ (molecule 7), which has a large number of electrons but a very small binding free energy. This exception can be straightforwardly rationalized. PCZ contains three main substituent groups connected to a central dioxolane ring. The nature of the bonding between these substituent groups and the central ring leaves PCZ with a relatively small conformational flexibility. This molecular rigidity limits the extent to which PCZ can reconfigure its substituent groups into an optimal pose to achieve a more favorable binding to the CNT surface.

**Figure 3:**
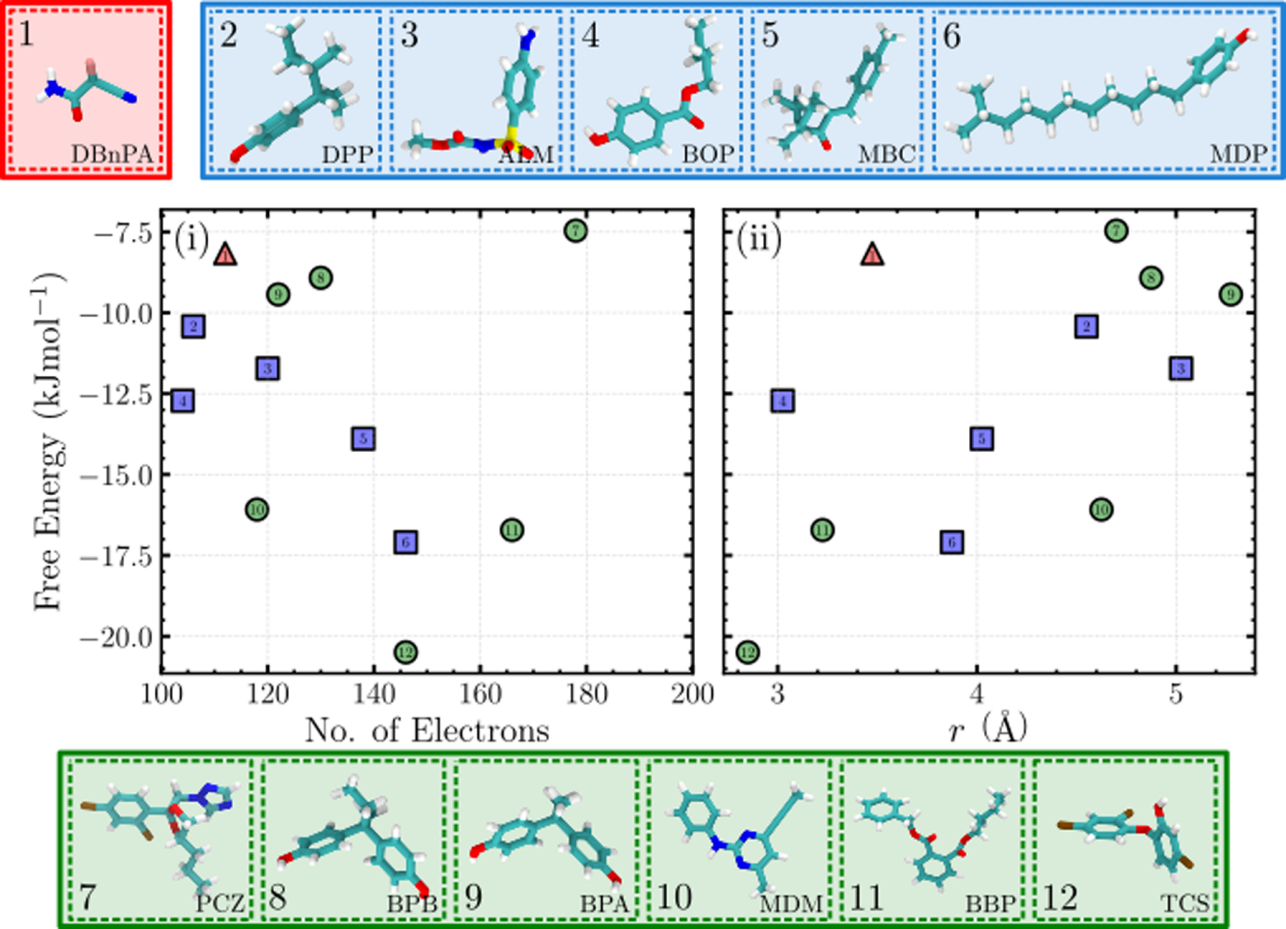
EDC–CNT interaction strengths (computed as the integral of the free energy surfaces in Figure 2) as functions of (i) the number of electrons in the molecule and (ii) the distance between the molecular COM and the CNT surface at the position of the minimum free energy. The points have been colored according to the number of aromatic groups within the molecule. The red triangular points correspond to molecules with no aromatic groups, of which only one was studied. The blue square points correspond to molecules with a single aromatic group. The green circular points correspond to molecules with two aromatic groups. The points in panels (i) and (ii) have been numbered to identify which point belongs to which molecule and each of the groups has been sorted from lowest (least favorable) interaction strength to highest (most favorable).

The minima in the free energy surfaces in Figure 2 all appear at different distances from the CNT surface. It is therefore reasonable to suggest that molecules that can come closer to the CNT surface will bind to it more strongly. To study this further, we found the positions of the deepest minimum in the free energy landscape for each of the studied molecules. The value of *r* corresponding to this minimum was then plotted against the binding free energy. This analysis is presented in Figure 3 (panel (ii)), which shows that while it is generally true that molecules whose centers of mass can approach closer to the CNT surface have higher binding free energies, there are some noteworthy exceptions. DBnPA (molecule 1), the only molecule studied which does not possess any aromatic groups, gets quite close to the CNT even though it does not strongly bind. This result is not surprising given how small this particular molecule is. Figure 3 (panel (i)) shows that small molecules with low electron counts have low binding free energies. However, small molecules such as DBnPA are able to position their COMs close to the CNT because they do not possess large substituent groups that hinder their approach to the CNT surface.

### Adsorption Structure

Investigating the flexibility of the molecules when analyzing these trajectories is important as molecular flexibility is known to impact the adsorption of organic contaminants on other adsorbent species.^126^ The position of the molecular center of mass relative to the frame of the molecule can fluctuate a great deal during the simulation. This can have quite a pronounced effect on the collective variable used in our simulations, especially for molecules with long alkyl chains. Figure 4 highlights the volume of space which was explored by the COMs of MDP (upper panel) and TCS (lower panel) during a simulation of the isolated molecule. To construct these figures, we performed 1 ns-long simulations of each molecule under vacuum. The six atoms in one of the aromatic groups in these molecules were fixed throughout these simulations, but no constraints were applied to the other atoms, so the trajectory explored many molecular configurations that were different from those shown in the figure. The red clouds of points in Figure 4 show the volume of space that the molecular center of mass explored during the simulation. It is important to note that while not considered when running the isolated simulations of these molecules, the presence of the CNT wall will restrict the volume of space explored by the molecular COM pictured in Figure 4.

**Figure 4:**
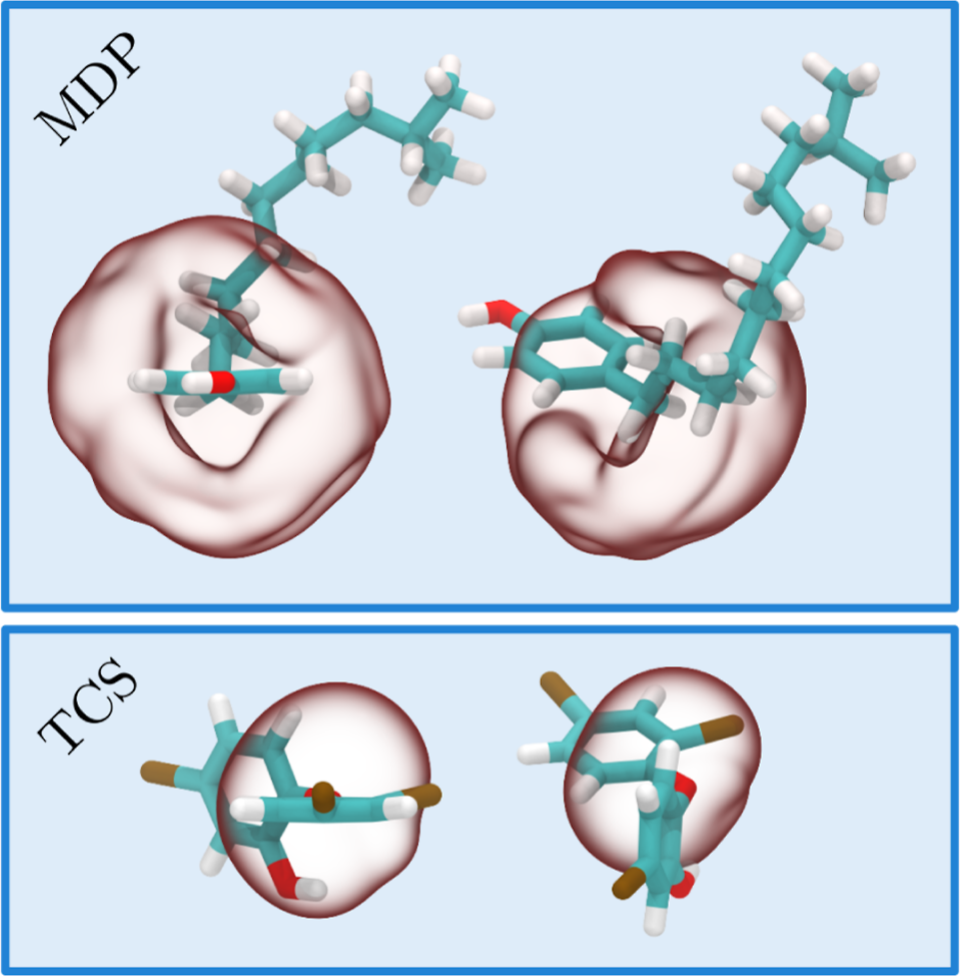
Schematic image of an isolated molecule of MDP (top panel) and TCS (bottom panel). The translucent red cloud represents the volume of space explored by the center of mass of the molecule in a 1 ns-long simulation.

The fact that the center of mass position fluctuates so widely in some of the EDCs renders incomplete any conclusions drawn solely from this analysis. Therefore, it is necessary to perform an additional analysis to gain insights into the binding conformers of these various molecules. For example, an analysis of the geometries that the molecules adopt when they bind to the CNT can be performed.

Organic molecules which are able to adopt configurations which maximize π-stacking interactions between the CNT surface and any aromatic substituent groups are adsorbed more favorably than molecules which cannot form these configurations. It is therefore reasonable to investigate the free energy of binding of the EDC molecules with regard to the orientation of any aromatic groups present in the molecular structure. To conduct this analysis, we computed the angle between the normal to the plane containing the atoms of any aromatic substituent groups (i.e., the six carbon atoms of a benzene ring) present in the molecule and the CNT surface normal. This angle was computed at every frame of the trajectory where the molecular center of mass distance to the surface, *r*, was within the first minimum in the free energy surface.

Figure 5 shows the results of this analysis, performed on the trajectories of TCS and BPA. These are two of the six studied molecules that contain two aromatic rings. TCS is the most strongly binding molecule studied, while BPA is one of the molecules that binds most weakly. Two histograms (one for each aromatic group) were then constructed from these angles. This analysis, performed for the other molecules with two aromatic groups, is presented as Figure S3 in the Supporting Information. The distributions presented in Figure 5 show that the normal vectors of the aromatic groups in the TCS molecules prefer to point in a direction that is parallel to the CNT surface normal. In other words, the aromatic groups in TCS lie flat on the surface of the CNT when the molecules bind. For BPA, by contrast, the distribution of this angle is close to uniform. When BPA binds to the CNT surface, the aromatic groups do not lie flat on the surface. This indicates that the aromatic groups in BPA are either not involved in the optimal adsorbed structure on the CNT surface or that due to the structural rigidity of the molecule, the adsorbed structure oscillates between configurations which involve either *R*_1_ or *R*_2_ lying flat on the surface. This, in turn, means that the interaction between the molecule and the CNT is rather weak.

**Figure 5:**
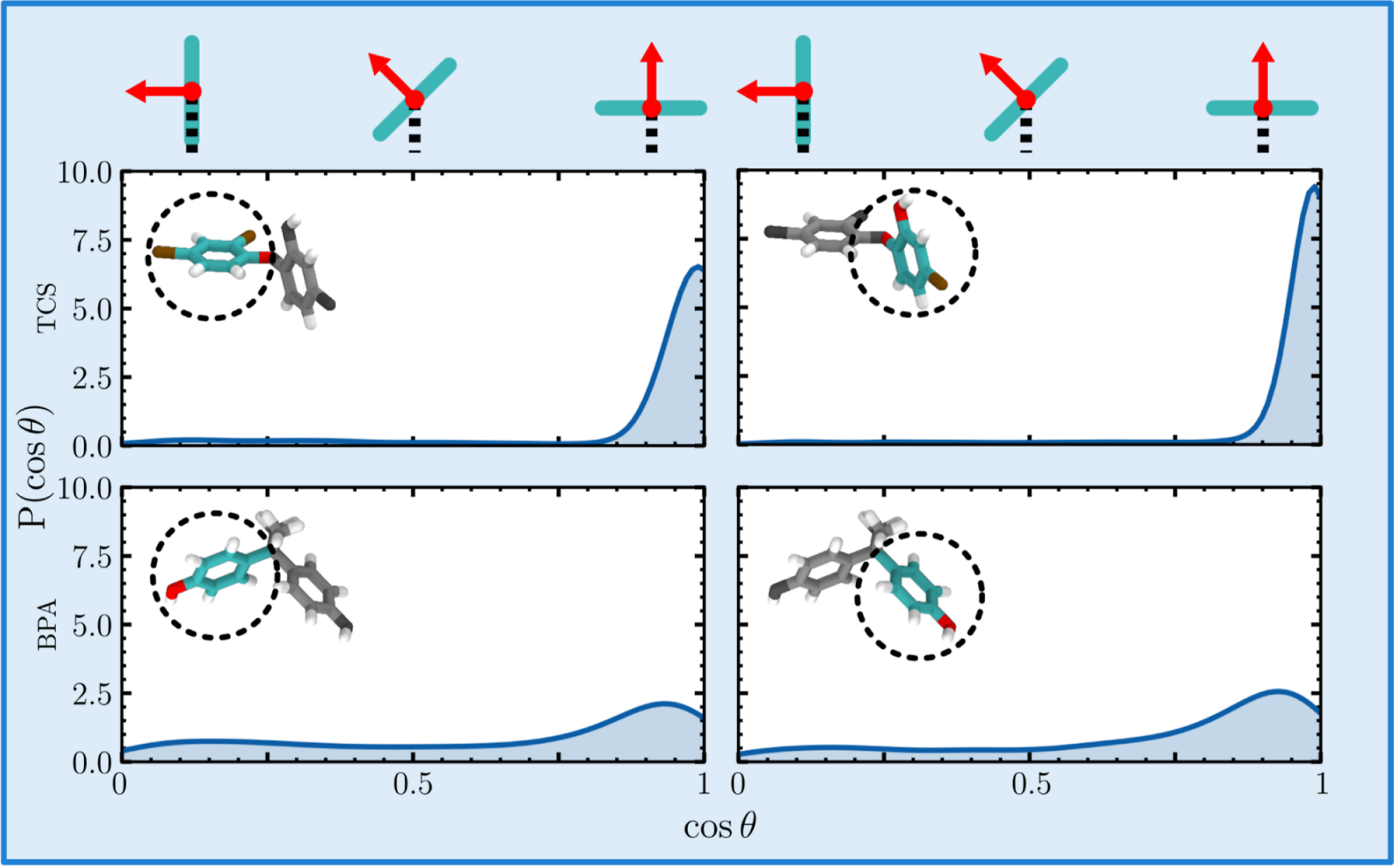
Probability density functions of the angles between the aromatic planes of *R*_1_ and *R*_2_ of TCS (upper left and right) and BPA (lower left and right) and the surface normal of the CNT. Illustrative images of how these vectors are arranged for cos θ = 0, 0.5, and 1 are provided at the top of the figure.

It is clear from the chemical structures of BPA and TCS that the two aromatic groups in TCS have much more conformational flexibility than the corresponding aromatic groups in BPA. The structure of TCS allows for the two aromatic groups to be in the same plane but the same cannot be said for BPA. Looking back at Figure 3, there are three molecules that contain two aromatic groups that bind strongly to the CNT (TCS, BBP, and MDM) and three that bind weakly (BPB, BPA, and PCZ). The molecules that bind weakly are all, like BPA, conformationally rigid. None of these molecules are able to achieve a configuration which enables the two aromatic groups to be coplanar. The three molecules that bind strongly all have a heteroatom joining the two aromatic groups. This heteroatom (either oxygen or nitrogen) confers rotational flexibility of the substituent groups onto the structure, which allows these molecules to adopt binding configurations that have coplanar aromatic groups. Adopting this confirmation upon approach to the CNT ensures a maximization of π-stacking interactions and strong binding to the CNT surface as a result.

The presence of a heteroatomic connection between two aromatic groups does not guarantee a high molecular flexibility. Short alkyl chains, either on the aromatic group or directly bonded to the heteroatom, can hamper the ability of a molecule to achieve an aromatic coplanarity. These effects are best observed by considering MDM and BBP, whose angular histograms are shown in Figure S3 in the Supporting Information.

MDM presents a markedly asymmetric adsorption behavior, particularly if compared to TCS. The pyrimidine group of MDM heavily prefers a planar overlap with the CNT surface, while the aniline group has little effect on the optimal adsorption configuration. This asymmetry arises from two factors: the higher rotational barrier around the C–NH–C bond compared to the C–O–C bonds of TCS or BBP and the strongly favorable π-stacking interaction between the CNT surface and the alkyne-substituted pyrimidine group. Any configuration involving both aromatic groups incurs an energetic penalty that cannot be recouped through surface interaction, resulting in the optimal configuration strongly preferring a planar overlap of pyrimidine over aniline. BBP, by contrast, can adopt coplanar configurations. However, the presence of both the benzyl group and the ortho butyl substituent on the group provides sufficient steric hindrances to make planar configurations less favorable.

Figure 6 shows the same analysis performed this time on the trajectories of MDP and DPP. This analysis was also performed on the other single aromatic molecules and is presented as Figure S4 in the Supporting Information. MDP and DPP are discussed here as the strongest and weakest binding molecules with a single aromatic group, respectively. It is clear from Figure 6 that an ability to preferentially adopt binding configurations where the aromatic groups are parallel to the CNT surface is not the only factor that contributes to strong binding. MDP is slightly more likely to be in the favorable configuration that has the aromatic group parallel to the CNT surface, but the difference is relatively marginal. In this case, the fact that MDP has many more electrons than DPP and that it also has a long hydrocarbon chain that is extremely hydrophobic likely has a more significant effect on the strength of binding than the preferential adoption of any particular pose upon binding to the CNT surface.

**Figure 6:**
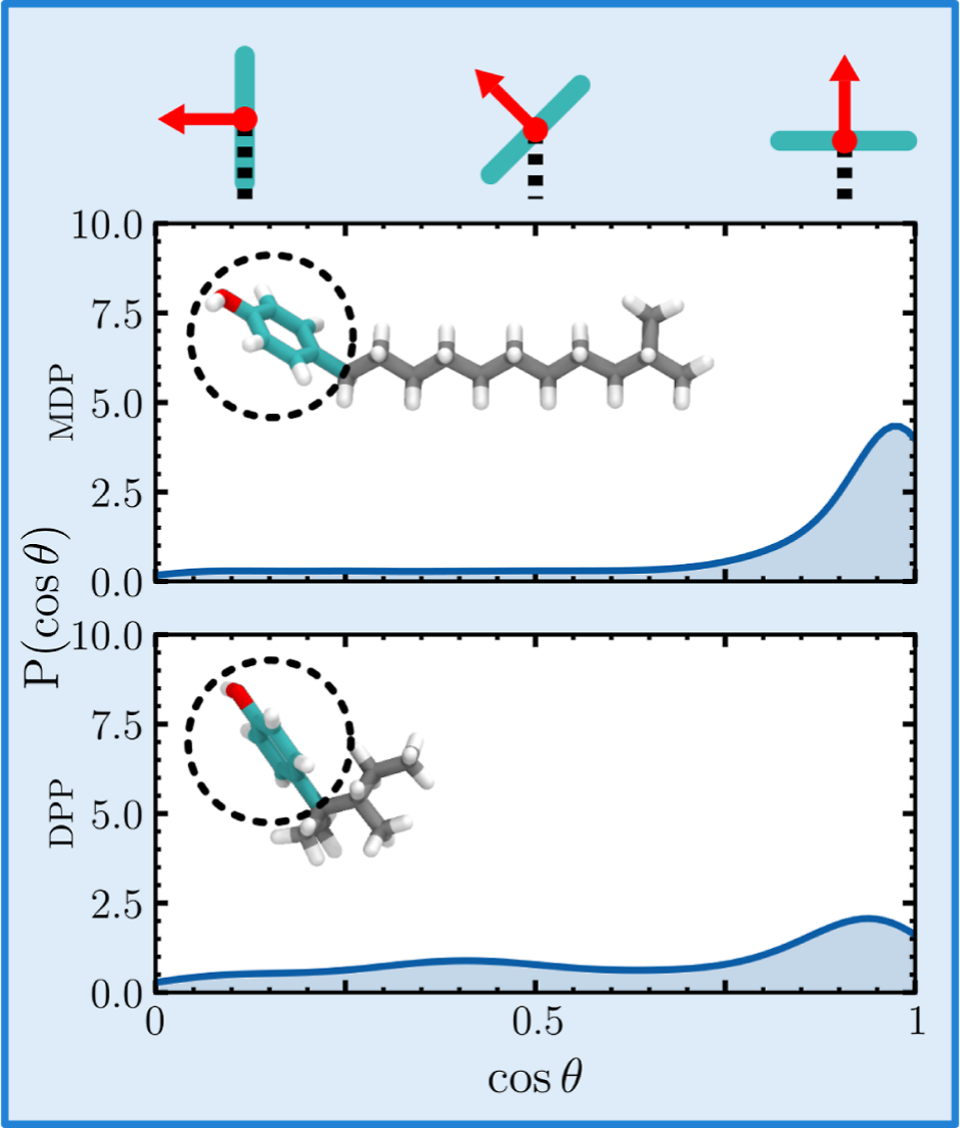
Probability density functions of the angles between the aromatic planes of the lone aromatics of MDP (upper panel) and DPP (lower panel) and the surface normal of the CNT.

## Conclusions

We have computed the binding free energies between 12 different EDCs and a CNT by using metadynamics. We find that all of the studied molecules bind preferentially to the CNT surface and that there is a reasonable degree of correlation between the number of electrons in the EDC and the binding free energy. In other words, larger molecules bind to the CNTs more strongly. However, there are some notable exceptions to this general rule. EDCs that contain aromatic groups bind strongly when they can adopt configurations in which the aromatic group lies flat on the CNT surface.

A close examination of Figures 5 and 6 demonstrates that the maximization of π-stacking interactions is particularly critical for strong binding. The EDCs we identified that bound the most strongly all had two aromatic groups connected to one another via a heteroatom. This increased rotational flexibility allows these molecules to adopt a conformation in which the two aromatic groups are coplanar. Molecules with two aromatic groups connected via a carbon atom cannot adopt configurations where the aromatic groups are coplanar and thus bind much more weakly.

When considering molecules that have only one aromatic group, we find that the number of electrons is much more clearly correlated with the binding strength and that the ability of these molecules to form configurations where the aromatic group is parallel to the CNT surface is far less critical. For the molecules studied in this work, we find that all of the molecules with only one aromatic group bind to the CNT much more strongly than rigid molecules with two aromatic groups.

In addition to molecular flexibility, the curvature of the nanotube may also have a significant effect on the free energy of adsorption, introducing further geometrical restrictions that affect the extent of π-stacking interactions. Further work exploring the relationships between surface curvature, molecular flexibility, and adsorption strength would greatly aid in the development of targeted molecular adsorption strategies.

Our work has demonstrated the insight that can be obtained by performing an exhaustive computer simulation using the same simulation protocols on multiple EDCs. We have demonstrated that such approaches illuminate both the binding free energies and the factors that lead to strong binding. In addition, we established a baseline upon which further real-world complexity may be considered. Future work could therefore extend the results presented within this study by considering the extent to which these adsorbents are fouled by other common aqueous organic materials. Performing similar studies with a range of EDCs, common competitive aqueous components, and adsorbates would provide a set of design principles and insights that could be used to optimize the processes for removing particular EDCs.

## Supplementary Material



## Notes

Baselining procedure used in the free energy analysis, free energy profiles of the EDCs not presented in Figure 2, construction of the planes used in the structural analysis of the adsorption process, and probability density functions of the molecules not discussed in this manuscript (PDF)

## Notes

A complete set of input parameters for each of the studied molecules, as well as example input files to complete both the molecular dynamics and metadynamics simulations, are available at https://github.com/sshepherd637/EDCsOnStuff. A permanent zenodo repository is also located at https://zenodo.org/records/20330898.
