# Supplementary material for: Thermodynamic and Structural Insights into Endocrine Disruptor Adsorption
Source: J Phys Chem C Nanomater Interfaces. 2026 Jul 6;130(28):9968–78. doi: 10.1021/acs.jpcc.6c02106 (PMC13383831; doi:10.1021/acs.jpcc.6c02106)
Supplement: Supplementary file 1 [file jp6c02106_si_001.pdf]

Thermodynamic and Structural Insights into Endocrine Disruptor Adsorption

Sam Shepherd* [1], Laura McWilliams [1], Oliver S. Cunningham [1], Helen Lubarsky [1], Gareth A. Tribello [1], Debra H. Phillips† [1], and David M. Wilkins‡ [1]

[1]Centre for Quantum Materials and Technologies, School of Mathematics and Physics, Queen's University Belfast, Belfast BT7 1NN, Northern Ireland, United Kingdom

Email: *sam.shepherd@qub.ac.uk †d.philips@qub.ac.uk ‡d.wilkins.qub.ac.uk

Contents

Free Energies **S1**

Adsorption Structure **S1**

Free Energies

Fig. S1 shows the average error in the free energy surfaces as a function of the block size used in our block averaging procedure. The dashed red line indicates the block size used to report our errors in this work and the associated error. This block size was 30000 data points which corresponds to 1.5 ns of simulation time.

The values of the fitting parameter c used in Eqn. 1., as well as the RMSE between the logarithmic functions and the free energy surfaces between 7 Å and 8 Å are reported in Tab. S1.

Fig. S2 shows the free energy profiles of BPB, BOP, DBnPA, MDM, BBP, and DPP.

Adsorption Structure

To construct the aromatic planes used in our analysis we used all of the aromatic atoms in the constituent group. The normal vector to each aromatic ring was computed using a least-squares plane fitting approach. The centre of mass (COM) of each ring was computed and used to translate the atomic positions to the COM frame. The covariance matrix of these positions was then diagonalised and the eigenvector corresponding to the smallest eigenvalue was found. This vector corresponds to the normal to the plane.

The angle was then computed by taking the dot product between the plane normal and the xy projection of the vector connecting the COM of the aromatic group and the COM of the CNT. A Gaussian kernel density estimation approach was then used to compute the probability density function (PDF) of the distribution of angles formed by the aromatic group on the surface. This was weighted by the corresponding weights from either metadynamics or un-biased simulation (in which case, the weight was equal to 1) depending on which trajectory the frame was obtained from.

Fig. S3 shows the PDF of aromatic overlap angles for the molecules with two aromatic groups not present in the main text, namely PCZ, BPB, MDM, and BBP. PCZ possesses three aromatic groups and hence requires three PDFs to fully capture its ability to form aromatic overlap at the CNT surface. Fig. S4 shows this same PDF for molecules with one aromatic group not present in the main text, namely BOP, MBC, and ALM. DBnPA does not contain any aromatic groups and this analysis was not performed for it as a result.

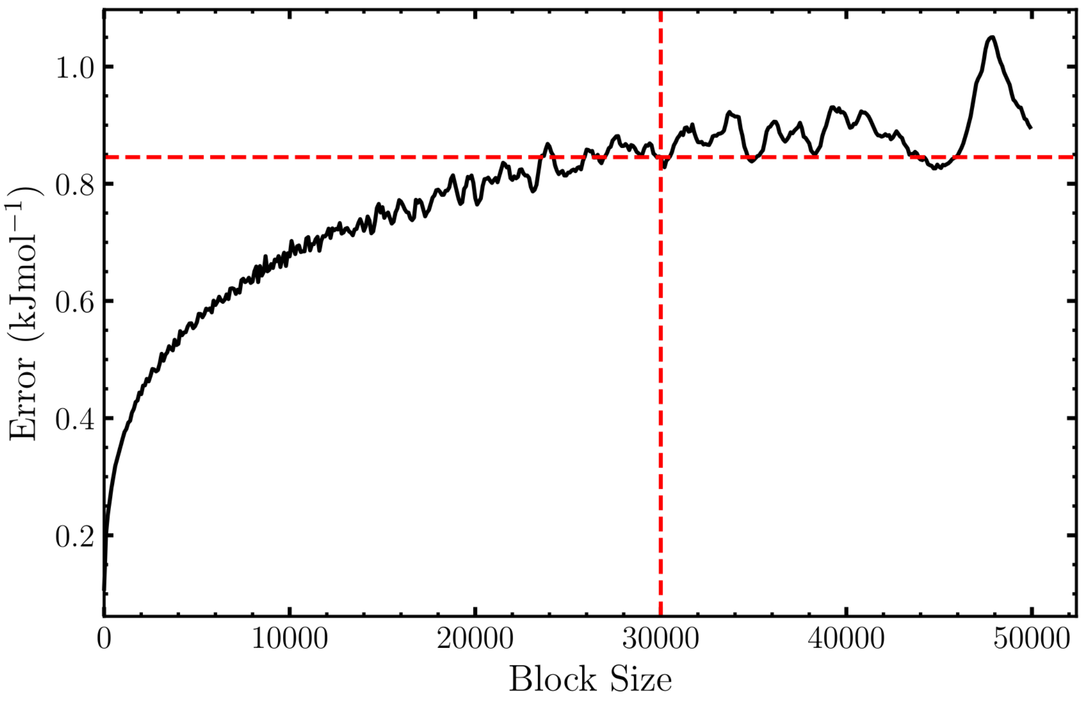

Figure S1: Error in the free energy as a function of block size.

Molecule	c	RMSE
TCS	38.038	0.2387
BPA	25.926	0.1966
PCZ	23.724	0.3508
ALM	28.428	0.5280
MDP	34.334	0.8834
MBC	30.831	0.8300
BPB	25.325	0.3673
BBP	33.934	0.8566
BOP	29.530	0.1599
DBnPA	24.525	0.4774
DPP	27.027	0.6838
MDM	33.233	0.8584

Table S1: Values of fitting parameter c and the corresponding RMSE for each of the studied molecules.

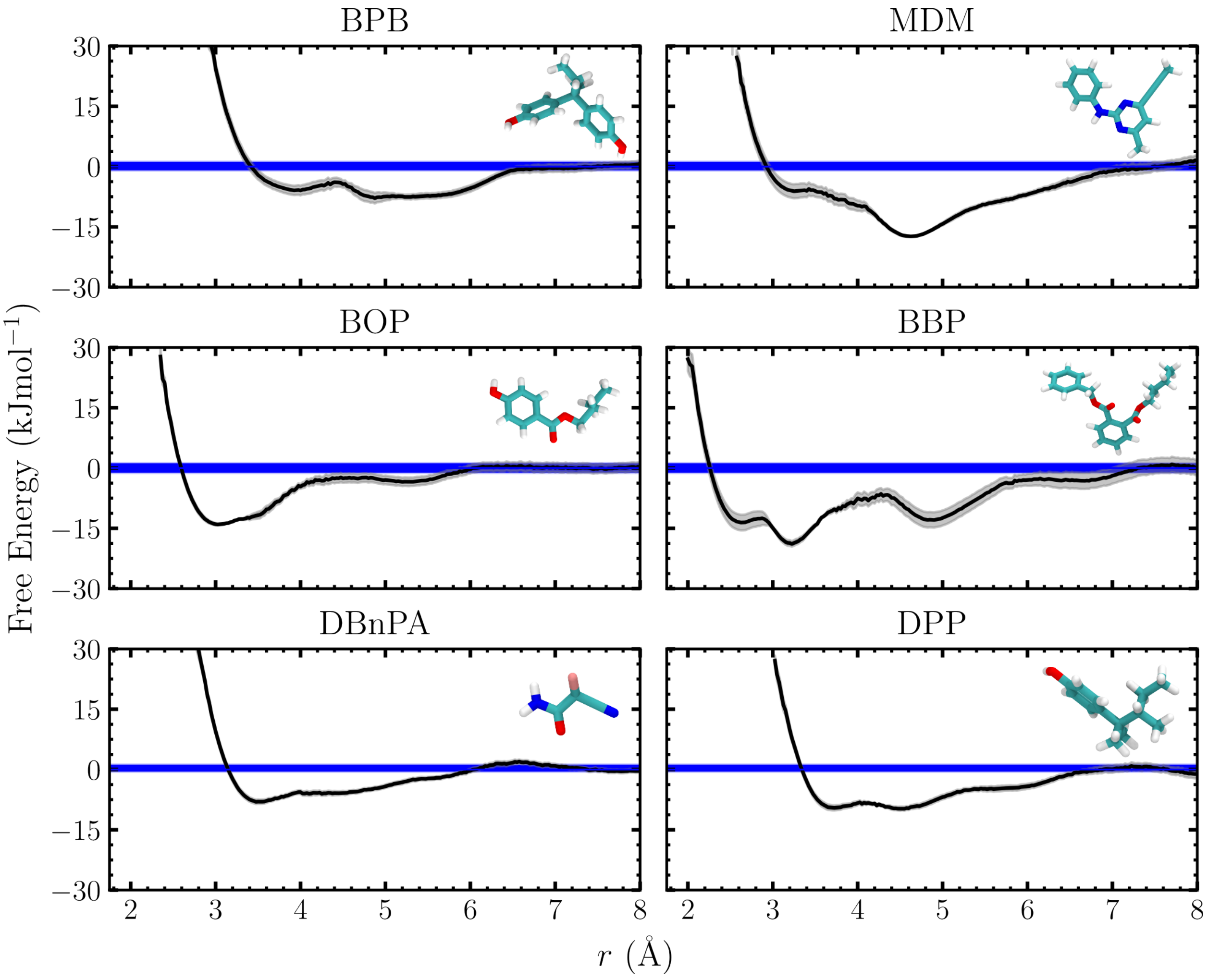

Figure S2: Free energy as a function of distance from the surface of the CNT. The error in the free energy profile is the gray area around the black line, while the error in the baseline is included as the lighter blue area around the solid blue line.

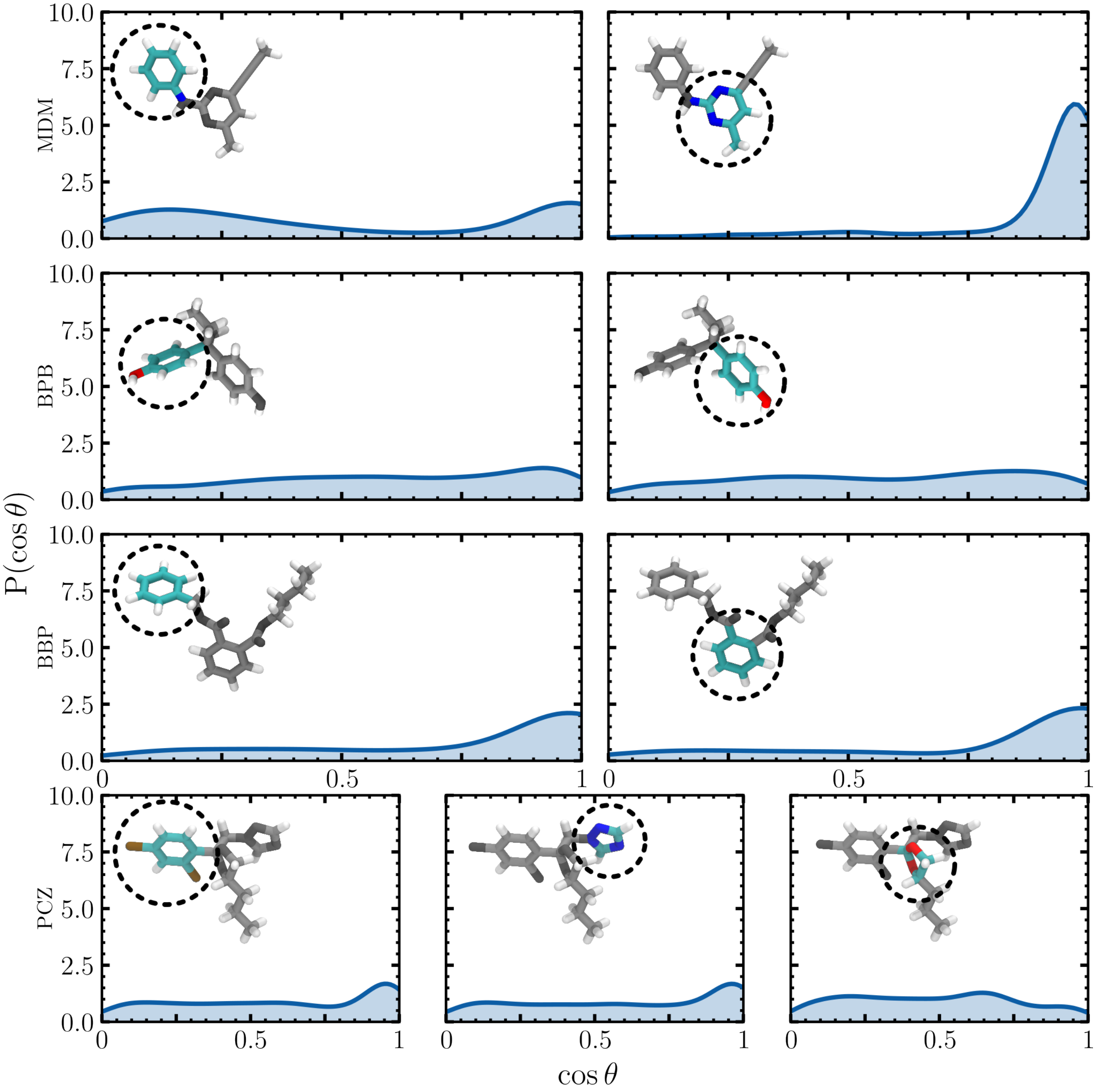

Figure S3: Probability density functions of the angles between the aromatic planes of R_1 and R_2 of MDM, BPB, BBP, and PCZ, and the surface normal of the CNT.

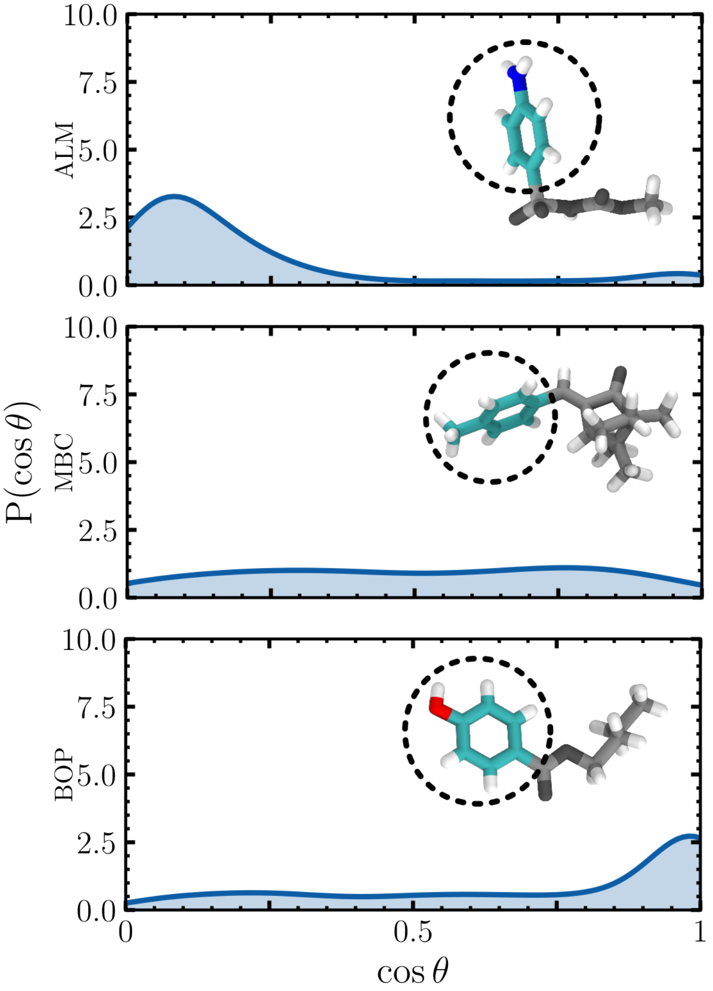

Figure S4: Probability density functions of the angles between the aromatic plane of ALM, MBC, and BOP, and the surface normal of the CNT.
